# Exploring the Acquisition of Social Communication Skills in Children with Autism: Preliminary Findings from Applied Behavior Analysis (ABA), Parent Training, and Video Modeling

**DOI:** 10.3390/brainsci14020172

**Published:** 2024-02-09

**Authors:** Daniela Bordini, Ana Cláudia Moya, Graccielle Rodrigues da Cunha Asevedo, Cristiane Silvestre Paula, Décio Brunoni, Helena Brentani, Sheila Cavalcante Caetano, Jair de Jesus Mari, Leila Bagaiolo

**Affiliations:** 1 Department of Psychiatry, Federal University of Sao Paulo (UNIFESP), Sao Paulo 04017-030, SP, Brazil; danibordini4@gmail.com (D.B.); anacmoya@gmail.com (A.C.M.); gracci.rc@gmail.com (G.R.d.C.A.); sheila.caetano@unifesp.br (S.C.C.); jamari17@gmail.com (J.d.J.M.); leila.bagaiolo@grupogradual.com.br (L.B.); 2 Human Development Sciences Program, Mackenzie Presbyterian University (UPM), Sao Paulo 01302-907, SP, Brazil; debruno46@gmail.com; 3 Psychiatry Institute, University of São Paulo (USP), Sao Paulo 01246-904, SP, Brazil; helena.brentani@gmail.com; 4 Gradual—Behavioral Intervention Group, Sao Paulo 05458-000, SP, Brazil

**Keywords:** autism spectrum disorders, applied behavior analysis, parent training, joint attention, video modeling

## Abstract

Social communication skills, especially eye contact and joint attention, are frequently impaired in autism spectrum disorder (ASD) and predict functional outcomes. Applied behavior analysis is one of the most common evidence-based treatments for ASD, but it is not accessible to most families in low- and middle-income countries (LMICs) as it is an expensive and intensive treatment and needs to be delivered by highly specialized professionals. Parental training has emerged as an effective alternative. This is an exploratory study to assess a parental intervention group via video modeling to acquire eye contact and joint attention. Four graded measures of eye contact and joint attention (full physical prompt, partial physical prompt, gestural prompt, and independent) were assessed in 34 children with ASD and intellectual disability (ID). There was a progressive reduction in the level of prompting required over time to acquire eye contact and joint attention, as well as a positive correlation between the time of exposure to the intervention and the acquisition of abilities. This kind of parent training using video modeling to teach eye contact and joint attention skills to children with ASD and ID is a low-cost intervention that can be applied in low-resource settings.

## 1. Introduction

Autism spectrum disorders affect around 1% of the population worldwide and can impact development, underscoring the need for early and adequate treatment aimed at target abilities [1]. Social communication impairments are a core feature required for the diagnosis of ASD and one of the earliest signs [2]. Moreover, eye contact and joint attention skills are the bases for social communication abilities and are essential to the early brain development process [3].

Most early treatment programs for children with ASD are based on the acquisition of eye contact and joint attention as prerequisites for other socio-communicative skills and spoken language [3]. Eye contact is considered a prerequisite skill for joint attention [4]. Eye contact is critical during infant development for learning gestural communication and referencing for cues and later acquisition of communicative behaviors; therefore, it is essential that it is learned so that more complex derived skills can be acquired more quickly and at the highest possible level [5,6]. Early intervention is critical to address early social deficits and avoid a cascade of impairments in learning and development [7,8]. For these skills to be acquired and generalized, it is essential that early, individualized, and intensive training is carried out and delivered in different settings (home, school, community, and/or clinic). One of the most effective treatments for ASD is applied behavior analysis (ABA) [9,10,11]. However, due to its cost and the need for specialist professionals, it is inaccessible to most of the population [12].

There are several evidence-based practices derived from ABA for teaching prosocial and social behaviors, such as differential reinforcement, naturalistic intervention, social narratives, discrete trial training (DTT), prompting, parental training, and video modeling [11,13,14]. Some of these strategies allow the use of interventions designed to avoid errors or incorrect answers, which is known as errorless learning. The contents are presented in a controlled and systematic way, being broken down into small “discrete” components in the case of DTT and into a hierarchy of “tips” in prompting [15]. ABA requires learned behavior to be recorded through direct and continuous observation by those delivering it, whether they are specialists or parents/caregivers [16].

Positive outcomes through ABA interventions require high intensity (number of hours per week) and long duration (in years, for example) [17,18]. However, it can be expensive for implementation in low- and middle-income countries [19]. Therefore, the use of parental training has emerged as a method to ensure the stimulation of children with ASD in a natural environment and to guarantee maximum exposure to ABA therapy [20]. Parents have many opportunities to train these skills throughout the day in different contexts, facilitating generalization. There are an increasing number of studies in the literature related to the effectiveness of parental training with respect to the main symptoms of autism, and the recommendations are varied, including different settings (home or community), formats (individual or group), methods (such as didactic instruction, discussions, and modeling) and type of intervention (focused or comprehensive) [11]. In addition, it is a particularity of behavior analysis to monitor the process of acquiring new repertoires, which is usually accomplished through a regular registry (e.g., a diary). Most studies do not collect data throughout the process, and for some types of analysis, this is very important [16].

Nevertheless, parental training studies based on ABA have been growing along with the importance and scope of treatment models using technological resources like video modeling [21,22,23,24,25,26]. Its use can promote the assistance of patients with ASD, making it easier for families to deliver effective care and decrease treatment costs.

There is a need to facilitate access to less costly evidence-based intervention for this population [27], especially for the most severe profile associated with intellectual disability. This is an exploratory study to assess the feasibility of an intervention model to acquire eye contact and joint attention in children with ASD by means of parental intervention using video modeling and to investigate the clinical factors related to the results.

## 2. Materials and Methods

This exploratory study comprised 34 children with ASD and ID who took part in the intervention program. The mean age was 4.79 (SD = 1.25) years old, ranging from 3 to 7, where 70.6% were males. The mean IQ was 59.90 (SD = 9.42), ranging from 49 to 75. Of the total sample, 73.5% of the children belonged to middle–low socioeconomic status, and 26.5% to middle–high.

In this study, we analyzed the intervention group (34 participants) of a broader project. It is, then, an excerpt from a pilot, multicenter, single-blinded 22-week randomized clinical trial of a parent-mediated intervention group using video modeling conducted between January and November 2014. Further methods detail of the wider study can be found elsewhere [28,29].

Participants of the wider study were sixty-seven families with children aged between 3 and 6 years and 11 months with ASD and ID and were enrolled in the RCT from three ASD centers: (i) the Social Cognition Clinic of the Federal University of Sao Paulo (TEAMM/UNIFESP); (ii) the ASD Program of the University of São Paulo (PROTEA/HC); and (iii) the Developmental Disorders Post-Graduation Program of the Mackenzie Presbyterian University (TEA-MACK). Randomization: sixty-seven families were randomized, where thirty-four families were allocated to the intervention group and thirty-three to the control group; however, one case of the control group had to be ruled out later due to a counting error regarding the ADI-R’s diagnostic criteria. In this study, only the intervention group (34 participants) was analyzed.

The inclusion criteria for this study were as follows: (i) children with an ASD diagnosis according to the Brazilian version of the Autism Diagnostic Interview (ADI-R) (ADI-R) [30], and a clinical evaluation by a multidisciplinary team of experts based on the Diagnostic and Statistical Manual of Mental Disorders (5th ed.; DSM-V) [31]—a medical evaluation (clinical observation) was carried out by a panel of psychiatrists from the three centers; (ii) children with an IQ between 50 and 70; and (iii) caregivers with at least eight years of schooling and able to read the intervention material. The exclusion criteria were children with uncontrolled epilepsy, those receiving intensive behavioral intervention (>10 h per week), children whose main caregiver had an ASD diagnosis, or those who did not have a DVD player at home. Before data collection, a parent or legal guardian signed a written informed consent. Regarding financial assistance, a full monthly allowance was provided for travel (round trip), as well as food for all participants, as help for attending all intervention and evaluation sessions during the training. This study was approved by the Ethical Committee of the Federal University of Sao Paulo (UNIFESP) under protocol number 19927213.4.1001.5505.

### 2.1. Professional Training and Development of Materials for Parental Training

All professionals involved in the evaluation were trained to apply specific tools and were blind to the outcome assessments. The supervisory team was composed only of behavior analysis experts and produced all the training material, as described in [28,29].

### 2.2. Measurements

1. The Brazilian version of the Autism Diagnostic Interview (ADI-R) was used as the diagnostic tool for all children at baseline. The ADI-R is a 93-item structured interview conducted with parents to measure four domains: reciprocal social interaction, communication, language, and patterns of behavior. The Brazilian version of the ADI-R was adapted and validated by Becker et al. (2012) [30]. A trained psychologist supervised the administration of all interviews.

2. *Associação Brasileira de Empresas de Pesquisa (ABEP)*: Socioeconomic level (SES) was assessed by a questionnaire developed by the Brazilian Association of Research Companies (ABEP) to evaluate the families’ socioeconomic level (SES) according to their purchasing power. This tool was applied at baseline and is one of the most used in Brazil. The scoring system considers, among other factors, the ownership (yes or no) and the number of household appliances, as well as the education level of the household head. Total scores determine the socioeconomic status of families, classifying them into five social classes (A, B, C, D, and E), where the higher the score, the higher the socioeconomic level. Social class A: scores 100–45; B: 44–29; C: 28–17; and D/E: 16–0. According to the estimated cost of the quantity of household appliances and level of education, ABEP reckons that the average family incomes for each socioeconomic strata (criteria updated by ABEP 2022) are as follows: A: BRL 21,826.74 or USD 4141.69; B1: BRL 10,361.48 or USD 1966.12; B2: BRL 5755.23 or USD 1092.07; C1: BRL 3276.76 or USD 621.77; C2: BRL 1965.87 or USD 373.03; and D/E: BRL 900.60 or USD 170.89 (with dollar conversion rate at BRL 5.27) [32].

The following two tools were applied (at week 28) before and after the intervention by independent evaluators who were blind to the outcome assessments. Parents were aware of the treatment assignment.

1. The Vineland Adaptive Behavior Scale—First Edition (VABS): A structured interview was conducted with the caregivers to assess the following domains: socialization, communication, daily living, and motor skills. Age-equivalent scores and standard scores (M = 100; SD = 15) are provided for each domain, and scores across domains can be combined to create an overall Adaptive Behavior Composite Score [33]. In this study, adaptive skills were assessed using age-equivalent scores because they show more sensitivity in young children with ASD and ID during the progression of the intervention [34].

2. The Snijders–Oomen Nonverbal Intelligence Test (SON 2½-7): A non-verbal measure of IQ comprising six tests (categories, analogies, scenarios, stories, mosaics, and patterns). This battery was standardized and validated for the Brazilian social and cultural context [35].

### 2.3. Parental Training by Video Modeling Based on ABA

The 34 participants were divided into three parental training centers and received 22-week, 90-min sessions. Two sessions were held with the children in order to correct, if necessary, the procedures to be followed; the remaining sessions were held with the caregivers. All procedures described below took place simultaneously and identically at the three sites.

The data were collected through Record Sheets, which were developed specifically for this study by the research team specialized in behavior analysis. It allowed caregivers to record all attempts to produce eye contact and joint attention behaviors, as well as the level of help used in each trial. Each training level used a different sheet. The sheet contained two blocks with space to record the details for 18 attempts, totaling 36 attempts in a day (examples of these sheets are contained in Supplementary Table S1). For each attempt, caregivers were instructed to record the level of help used. The levels followed a prompting hierarchy starting with a high level of prompting, which included full physical prompt (FPP), partial physical prompt (PPP), gestural prompt (GP), and independent (I).

Each parental training session was organized as follows: (1) the video was presented to the groups of parents; (2) the previous week’s record sheets were analyzed and checked; (3) the DVD and record sheet for the next level of training were handed, out or doubts about the videos were clarified with the parents through the previous videos; and (4) participants signed the attendance list at the end of the session. Any caregiver who had missed the previous week’s session was given the audio–visual material and the record sheets in addition to that week’s material.

The ABA-specialized professionals produced 15 videos for this clinical trial. With respect to the video modeling methods applied in this study, we devised structured and hierarchical prompts to be taught to the parents. The first four videos offered the theoretical and practical basis for the training itself. All of them contained objectives and descriptions of procedures, instructions to complete the record sheets, and how to apply the activities to the children. Family members were instructed through video modeling (video 3) to contingently reinforce the production of target responses (eye contact and joint attention) with possible reinforcers. This was explained within each video to parents, who were also asked to regularly assess for potential reinforcers to use with their children.

The remaining 10 videos contained a sequence of prompts to teach eye contact and joint attention; the last one was a review. At each session, the families received a copy of the video shown that day to practice at home. More details about video contents were described elsewhere [28,29].

The parents were instructed to watch the DVD at home and apply the procedures twice a day, at different times, during the 22 weeks, from Monday to Sunday. The caregiver registered on the record sheet the level of help (FPP, PPP, GP, or I) used in each attempt.

Participants received food vouchers and transportation allowance to travel from their homes to each center throughout this study. All participants were from greater Sao Paulo (city of São Paulo and surrounding areas). On average, it took 1 to 2 h for each participant to get to the collection locations by public transport. This work was supported by the São Paulo State Financing Agency—FAPESP—under a special agreement with the Maria Cecília Souto Vidigal Foundation (grant number 2012/51584-0). NGO Autismo and Realidade supported the administration of the scholarship.

**Figure 1:**
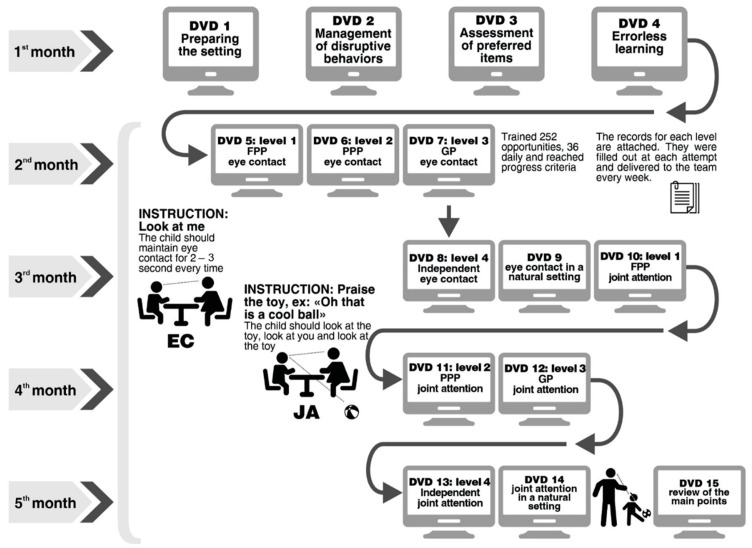
Parent training summary.

The parental training methodological procedures are summarized in Figure 1.

The initial training was on acquiring eye contact skills, which are considered a prerequisite for joint attention acquisition, and focused on triangulation and eye-gazing.

The DVD procedures were related to errorless learning and DTT practices with essential components such as reinforcement and prompts to acquire both skills. The most-to-least (MTL) prompt (starting with a high level of prompting) was selected for the acquisition phase due to the sample being composed of ASD children with intellectual disabilities. The prompting hierarchy was as follows: full physical prompt (FPP) → partial physical prompt (PPP) → gestural prompt (GP) → independent (I). This method used a “fading process”, starting with a physical prompt, such as a soft touch on the face, then a gesture, for example, indicating where the eye should be directed until the ability became independent.

During this training period, parents were given the task of performing two blocks of nine trials twice a day so that the child was exposed to 36 daily opportunities to practice the skills. There was a two-minute interval between each block. The parent/guardian practiced the activity with the child throughout the week, and after mastering the attempts at level 1 (FPP), the family member received a level 2 (PPP) video and record sheet and continued until the child could independently perform these behaviors. Therefore, caregivers first learned about FPP and were instructed to register 36 attempts per day of this level of support. Then, they learned about PPP and registered which type of prompt the child needed for each attempt. From this point on, as caregivers learned about different types of prompts, they were instructed to try to give less support and offer higher levels of support only as needed. Progression criterion: completing three blocks of consecutive training with at least eight out of nine correct responses per block using the programmed prompting hierarchy (PPH). Finally, children who were not able to acquire independent eye contact after their parents finished DVD 8 were given an extra week to train these skills. During this period, all children who had acquired independent eye contact stopped registering in the record sheets.

Data analyzed in this study include only the record sheets for DTT practices with the most-to-least prompt strategy.

### 2.4. Statistical Analysis

The outcome measures of this study were recorded as follows: (i) eye contact—full physical prompt, eye contact—partial physical prompt, eye contact—gestural prompt, and independent eye contact, as measured by the eye contact record sheets; and (ii) joint attention—full physical prompt, joint attention—partial physical prompt, joint attention—gestural prompt, and independent joint attention, as measured by the joint attention record sheets. Sex, age, age equivalent scores of the Vineland socialization domain, IQ measures, training (total number of completed record sheets for each family, used as a proxy of adherence), and time (days of training) were treated as covariates.

Four generalized estimating equations (GEEs) were used to evaluate the four graded measures of both eye contact and joint attention (i.e., full physical prompt, partial physical prompt, gestural prompt, and independent). The GEEs’ working correlation matrix used was the first-order autoregressive with a robust estimator. The model underlying the GEEs was linear because the scores for each one of the four assessed parameters were considered continuous variables. All the covariates were added to the model. All the models were run using SPSS version 24 [36], and the adopted significance level was 0.05. In this study, we will be using only the data of the intervention group because our goal was to analyze the skill acquisition progress in the experimental group.

## 3. Results

The data collected from the eye contact record sheets and joint attention record sheets provided the outcome measures that will be described in the following paragraphs.

The means, the standard deviations, and the minimum and maximum values of IQ, ABC total score, and Vineland standard scores of communication and socialization are described in Table 1.

**Figure 2:**
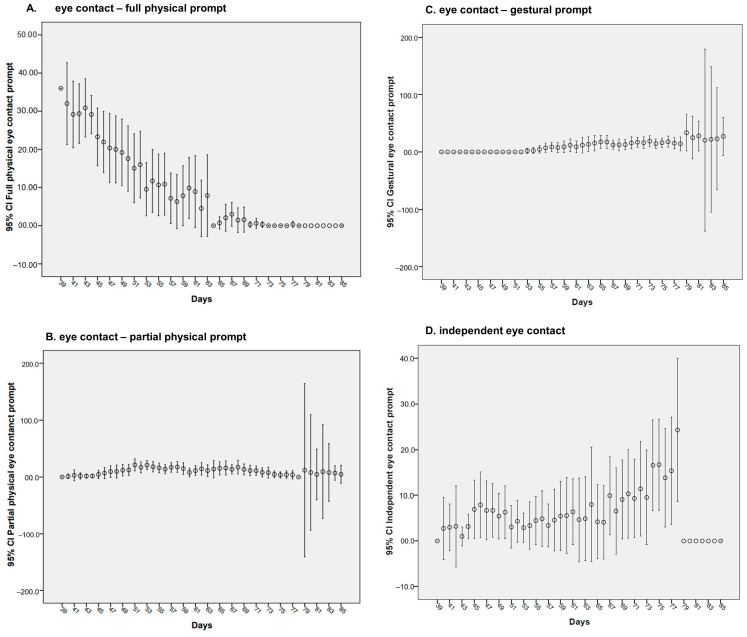
Trajectory for eye contact acquisition.

Figure 2 depicts the means of attempts (with 95% confidence intervals) across time (days) for the four outcomes: full physical prompt (FPP), partial physical prompt (PPP), gestural prompt (GP), and independent (I) in respect of eye contact. The number of attempts could vary from 0 (if the family did not apply the protocol on a given day) to 36 (the maximum number of attempts per day).

Table 2 shows the effects of all covariates on the four assessed outcomes. Among the four measures, there was, on average, a reduction of 0.862 (*p* = < 0.001) in daily total eye contact—full physical prompts count, meaning as time goes by, there is a reduction in the prompts count. Moreover, there was an increase in the daily average number of eye contact gestural prompts count of 0.537 (*p* = 0.029). Regarding sex, we observed that for eye contact—partial physical prompts count, males required, on average, five eye contact partial physical prompts count less than females across the whole study. For the other outcomes, there were no statistically significant differences between the sexes.

There was an association between the training received (number of completed record sheets per family) and the number of partial physical prompts. As the number of sections increases, the frequency of partial physical prompts also increases (Β = 0.132, *p* = < 0.001).

IQ and age were positive statistically significant predictors of the number of gestural prompts (Β = 0.442, *p* = < 0.001) and (Β = 0.277, *p* = < 0.001), respectively.

**Figure 3:**
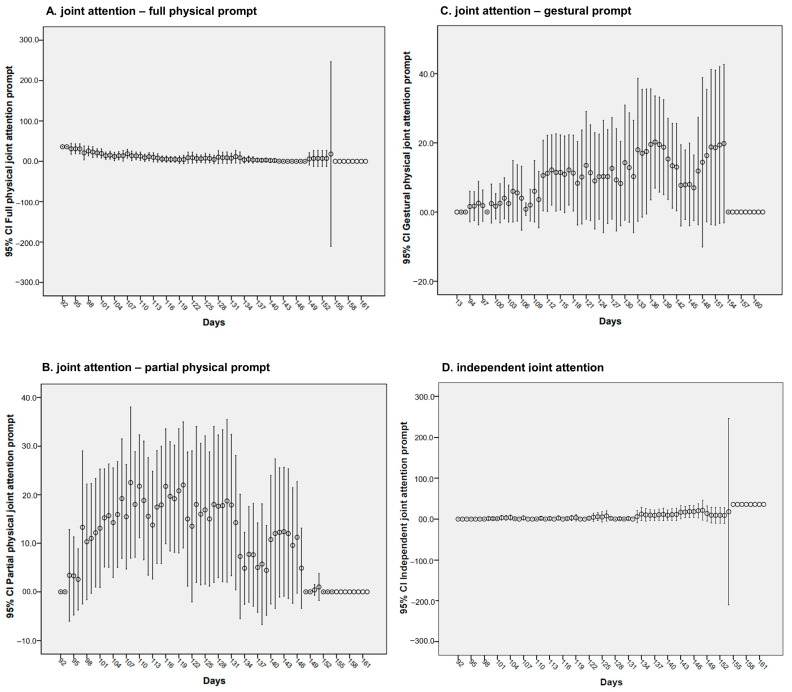
Trajectory for joint attention acquisition.

Figure 3 depicts the means of attempts (with 95% confidence intervals) across the intervention period (days) for the four outcomes regarding joint attention. The number of attempts could vary from 0 (if the family did not apply the protocol on a given day) to 36 (the maximum number of attempts per day).

Table 3 shows that the number of joint attention full physical prompts count decreased by 0.4 daily over the intervention period (Β = −0.449, *p* = < 0.001), and the number of independent joint attention increased by 0.251 daily (Β = 0.251, *p* = < 0.001). Other statistically significant effects were the VABS scores (Β = 0.218, *p* = 0.006), meaning that for every one-unit in the VABS score, there is an increment of 0.218 prompt count increment, on average, on independent joint attention. For IQ (Β = 0.354, *p* = 0.008), meaning the higher the IQ, the higher independent joint attention performance and age (Β = 0.242, *p* = 0.005), which means that the older the child, the higher independent joint attention prompt count. In other words, the higher the VABS scores, the more independent behavior was observed. In the same way, we found that the higher the IQ, the greater the independence, and, lastly, the older the children, the more independent they were. The number of joint attention partial physical prompts and gestural prompts was not statistically significant (Β = −0.066, *p* = 0.258 and B = 0.162, *p* = 0.107, respectively). 

## 4. Discussion

### 4.1. Parental Intervention in Eye Contact and Joint Attention Acquisition

In this exploratory study, we evaluated the process of children with ASD and ID acquiring eye contact and joint attention through video modeling in a parental intervention group. Overall, we found a progressive reduction in the level of prompts required over the intervention period for both target skills—eye contact and joint attention.

This may indicate that parents can be transformative agents and have a significant role in the treatment of their children with ASD. Their ability to implement therapies (under experienced supervision) and to achieve changes in their children’s abilities have been increasingly documented in recent years [12,20,37,38,39,40,41,42,43,44]. This is particularly important in situations in which there is a lack of trained professionals [12,20].

During the process of acquiring the skill of eye contact, there was a gradual, statistically significant reduction in the need for physical prompts and an increase in gestural prompts. This can be regarded as a reflection of progress, as gestural prompts are more difficult to execute and less invasive than physical prompts. It allows the child to respond following only a gesture and, therefore, generates an increase in independence. As our sample comprised children with ASD and ID, the most-to-least procedure was especially useful in teaching skills and maximizing learning capacity [45]. The Gulsrud hypothesis states that early interventions focused on prelinguistic and gesture repertoires for children with ASD may change the joint attention trajectory over time [46]. In this sense, the intervention model described in this study seems to produce this type of long-term benefit.

Another finding in this study was a positive association between intervention and joint attention ability. Although ABA is considered the most effective treatment for children with ASD, there is still a great deal of debate about the ideal treatment dosage, that is, its intensity and duration, to achieve the best results. Linstead et al. (2017) assessed the influence of these two factors separately on eight main outcomes (academic, adaptive, cognitive, executive function, language, motor, play, and social skills) in children with ASD. A solid linear relationship between skill acquisition and both treatment duration and intensity was demonstrated for all the factors, although the dose–response relationships were greater in the academic and language domains [17].

Families can have an important role in increasing treatment dosage, with many developmental and behavioral studies reinforcing the central role of the family in the treatment of children with ASD [11,27,47,48,49,50]. Parental training increases the number of treatment hours the children receive by incorporating stimulation strategies into routine activities. This decreases the need for a sizeable, specialized team, making treatment less costly and easier to apply. These studies, like ours, show the potential for improving the developmental trajectory of children with ASD by involving parents at an early stage in the use of stimulation techniques to develop socio-communicative engagement [27].

### 4.2. Association between Intervention, Joint Attention, and Predictors of Prognosis

Our findings also show that the acquisition of independent joint attention was positively associated with higher IQ levels, older age, and better social functionality. Overall, the most documented predictors of better prognosis in children with ASD are IQ level, social functionality, and communicative ability [51,52,53,54,55,56,57].

It is known that individual abilities at baseline predict, to some extent, outcomes in early intensive interventions in ASD [58]. Higher initial cognitive levels and fewer measured early social interaction deficits promote better acquisition of skills in developmental areas, receptive and expressive language, and play skills. Moreover, the cognitive functioning profile of children with ASD before intervention programs predicts the participants’ growth rates [59]. A recent clinical trial with parental training in children with ASD and disruptive behaviors identified levels of cognitive potential (an IQ greater than or less than 70) as one of the determining factors for functional improvement [60]. One of the most consistent findings in longitudinal studies about childhood predictors of better outcomes in ASD adulthood is intellectual functioning [52,55].

Despite the great number of studies in the literature showing better results from early interventions [34,58,61,62], we found a positive association between age and joint attention acquisition. This finding is not in line with most of the literature. We hypothesize that this may be explained because older children are more likely to learn due to the neuronal maturation process itself [63] or that they may benefit from repetition of the experiences in their longer lives. As prompting is a widely used technique with inclusive evidence for improving children’s joint attention in this age group [11,64,65,66], we think it is hasty to extrapolate the results of this study with a small sample. Further studies need to be undertaken to better understand the issue of age.

In our study, males required, on average, fewer partial physical prompts with respect to eye contact than females. Findings on this subject are contradictory, with some studies reporting that ASD cases are more severe in girls than boys while not confirming these results [67,68,69,70,71,72]. However, there was no statistically significant difference between sexes in our data concerning initial IQ. Further investigation is required to understand if sex, regardless of severity, can impact skill acquisition.

### 4.3. Main Aspects of This Intervention: Video Modeling, Record Sheets, and Short-Term Parental Intervention

According to Wetherby et al. [27], the main gaps in the evidence base of interventions for toddlers with ASD were with respect to the following: (1) the level of intensity needed to change the development trajectory. Current recommendations are for intervention of 15–25 h per week, but this is unfeasible for any public system, especially in LMIC; (2) the need to increase the number and intensity of parental training sessions to achieve results in children, thereby reducing the need for more intensive interventions in the future; and (3) reliable and meaningful outcome measures for toddlers with ASD, particularly with reference to registering changes in the natural environment. We tried to address all these factors in this study. By using parental training, we can increase the level of intensity of intervention at a low cost while also addressing the need to train parents to achieve better results in children. Record sheets may represent direct assessments to measure outcomes in the children in their natural environment if observer reliability is assessed. The advantages of training parents in group sessions are that they provide an opportunity for participants to share their experiences and offer emotional support to people with similar problems, in addition to also optimizing the use of resources and time of the specialized professionals. 

Recorded material can be a valuable resource to help families of children with ASD, especially in the current pandemic context. In the last few years, the advance of videos and other techniques has been used to offer greater support and information to parents, especially those who do not have easy access to services [11,21,23,24,25,26,42,50]. In 2016, a review of the use of video modeling demonstrated preliminary positive findings in the treatment of people with ASD but suggested that more extensive studies with more cases were needed to confirm the effectiveness of this technique [73].

The use of record sheets to constantly monitor the participants’ performance is standard practice in ABA research, but usually only in single-subject studies or a series of few cases. This study used a randomized clinical trial, a research methodology traditionally used with larger samples and pre- and post-intervention measurements. This allowed the use of methods already tested in single case/small groups to be assessed in larger groups to develop evidence-based practices and answer a number of questions regarding the methodology for this patient profile [74]. The five practices used in this study—discrete trial training, parent-implemented interventions, prompting, reinforcement, and video modeling [11]—are all practices that may be implemented in low-income families and the public health sector. This gives a more robust scientific and methodological relevance to the findings, increasing confidence in the use of this type of therapy for this profile of participants.

Finally, most studies stress the need for behavioral interventions with a long duration and a great number of hours in more severe cases of ASD, especially with associated ID [59,75,76]. One of our study’s strongest points is to demonstrate that children with ASD and ID may be able to acquire the learned behaviors through a relatively short-term parental intervention even though their children had IQs in the lower range. 

### 4.4. Limitations

This study underscores the importance of exercising caution in the interpretation of its preliminary findings, given the following limitations. The absence of direct evaluation tools such as ADOS, the lack of an active control group, and the reliance on a relatively small sample size highlight the need for prudence in generalizing the results. Additionally, the absence of interobserver agreement data and the reliance on analyzed sheets for parental information further emphasize the importance of approaching the findings with a degree of caution.

### 4.5. Future Directions

A future possibility of advancement for this study could include the use of a much larger sample size with repeated measures in a longitudinal design, which would allow the consideration of alternative statistical approaches [77]. For example, to account for both within-subject and between-subject variability by adopting models that embody individual-specific random effects. A generalized mixed-effects model (also known as a hierarchical or multilevel model) could be more suitable. This model allows for the inclusion of random effects, which can capture individual differences in the response variable [78,79].

Finally, future research incorporating an investigation of neurobiological aspects, such as neuroimaging and eye tracking, may help understand neural mechanisms underlying specific acquisitions [80]. Research examining which individual characteristics can moderate or predict treatment response is still lacking. A better understanding of which child characteristics can be associated with more significant gains may help clinicians choose more personalized and targeted therapeutic strategies [81]. Moreover, investigating the neural and physiological correlates of joint attention and eye contact in response to parental interventions could contribute to a more comprehensive understanding of the intervention’s impact on the developing brain in children with ASD and ID [82].

## 5. Conclusions

In summary, this original exploratory study, conducted in a family-centered group format with individuals diagnosed with autism spectrum disorder (ASD) and intellectual disabilities (IDs) in need of treatment, yielded encouraging results. However, it is important to interpret these findings cautiously. Given its relatively short-term nature and cost-free approach, the intervention holds promise for application in low- and middle-income countries and communities with limited access to health services, potentially making a valuable contribution to enhancing public healthcare. Yet, further research and long-term assessments are warranted to validate and refine these promising initial outcomes.

## Figures and Tables

**Table 1:** Sample profile characterization.

Measures			Minimum/ Maximum	Mean	SD	Mean	SD	
		N	Baseline	Baseline	Baseline	Post	Post	
Outcomes	ABC total score	34	46/139	94.94	22.29	70.55	22.74	
	IQ	34	50/76	60.21	9.08	66.65	19.47	
	Vineland communication standard score	27	32/65	47.48	7.78	45.86	9.41	
	Vineland socialization standard score	27	37/92	58.89	13.37	56.04	11.09	
Covariates	Age Socio-economic level	34	3/7	4.76	1.23	-	-	
		34	15/37	24.91	5.32	-	-	
	Hamilton’s Caregiver	34	0/23	10.18	7.86	-	-	
	ADI Sex (male)	32	31/60	47.53	6.61	-	-	
		24	-	70.60%	-	-	-	

ABC, Autism Behaviour Checklist; IQ, intelligence quotient; SD, standard deviation; ADI, Autism Diagnostic Interview. This table is based on Bordini et al. (2020) [29] Table 1, from which the sample of this study is based.

**Table 2:** Description of generalized estimating equations models for eye contact.

Covariates	Eye Contact Full Physical Prompt				Eye Contact Partial Physical Prompt				Eye Contact Gestural Prompt				Independent Eye Contact			
	Estimate	95% Confidence Interval		*p*	Estimate	95% Confidence Interval		*p*	Estimate	95% Confidence Interval		*p*	Estimate	95% Confidence Interval		*p*
Male	−3.255	−9.297	2.786	0.291	−5.562	−8.684	−2.440	<0.001	1.636	−1.940	5.123	0.370	5.957	−0.290	12.204	0.062
Vineland	−0.107	−0.281	0.066	0.225	0.010	−0.161	0.180	0.911	0.028	−0.186	0.242	0.801	0.135	−0.303	0.572	0.547
IQ	−0.195	−0.532	0.142	0.256	−0.243	−0.514	0.029	0.080	0.442	0.231	0.654	<0.001	0.059	−0.334	0.452	0.770
Age	0.038	−0.135	0.210	0.668	−0.174	−0.377	0.028	0.091	0.277	0.140	0.415	<0.001	−0.076	−0.485	0.334	0.717
Training	−0.015	−0.156	0.126	0.838	0.132	0.072	0.193	<0.001	−0.076	−0.233	0.080	0.339	0.055	−0.172	0.281	0.635
Time	−0.862	−0.987	−0.736	<0.001	0.079	−0.069	0.228	0.294	0.537	0.340	0.734	<0.001	0.249	−0.051	0.549	0.104

**Table 3:** Description of generalized estimating equations models for joint attention.

Covariates	Joint Attention Full Physical Prompt				Joint Attention Partial Physical Prompt				Joint Attention Gestural Prompt				Independent Joint Attention			
	Estimate	95% Confidence Interval		*p*	Estimate	95% Confidence Interval		*p*	Estimate	95% Confidence Interval		*p*	Estimate	95% Confidence Interval		*p*
Male	1.415	−4.206	7.035	0.622	1.379	−6.053	8.810	0.716	0.911	−4.822	6.644	0.756	−1.820	−5.574	1.934	P
Vineland	−0.057	−0.349	0.236	0.705	0.001	−0.316	0.319	0.993	−0.007	−0.277	0.262	0.957	0.218	0.061	0.375	0.342
IQ	−0.222	−0.723	0.280	0.386	−0.471	−0.962	0.020	0.060	0.282	−0.200	0.763	0.252	0.354	0.093	0.614	0.006
Age	−0.023	−0.344	0.298	0.888	−0.246	−0.495	0.004	0.054	0.057	−0.245	0.359	0.712	0.242	0.072	0.412	0.008
Training	−0.022	−0.202	0.158	0.811	0.022	−0.175	0.219	0.825	0.047	−0.138	0.233	0.616	0.080	−0.028	0.188	0.005
Time	−0.449	−0.584	−0.315	<0.001	−0.066	−0.181	0.049	0.258	0.162	−0.035	0.359	0.107	0.251	0.046	0.457	0.017

## Data Availability

Data is contained within the article and Supplementary Materials.

## Supplementary Materials

The following supporting information can be downloaded at: https://www.mdpi.com/article/10.3390/brainsci14020172/s1, Table S1. Description, target behaviors and duration of the 14 videos.
