# Supplementary material for: Exploring the Acquisition of Social Communication Skills in Children with Autism: Preliminary Findings from Applied Behavior Analysis (ABA), Parent Training, and Video Modeling
Source: Brain Sci. 2024 Feb 9;14(2):172. doi: 10.3390/brainsci14020172 (PMC10886593; doi:10.3390/brainsci14020172)
Supplement: Supplementary file 1 [file brainsci-14-00172-s001.zip › brainsci-2663967-supplementary.pdf]

The Supplement Table S1 summarizes the content, duration, and YouTube links of the 14 videos used in the parental intervention program.

Supplement Table S1. Description, target behaviors and duration of the 14 videos.

Video	Description and Target Behaviors	Duration (min)	Youtube link
1. Preparing the setting.	Parents were instructed in how to organize the therapy room (chosen place where the skills would be taught). Instructions included what could stay and what should be removed from the setting.	3min31s	https://www.youtube.com/watch?v=uNLhW5O8-BU&list=PLbgAY2-LxYGq4EvICE_QDd-r9j_a_Nn4I&index=2
2. Management of disruptive behaviors	Instructions about procedures to control the child's behaviors, minimizing inappropriate behaviors (e.g.: leaving the table without permission) and maximizing appropriate behaviors (e.g.: sitting).	22min23s	https://www.youtube.com/watch?v=l_Q5WX7QaYI&list=PLbgAY2-LxYGq4EvICE_QDd-r9j_a_Nn4I&index=3 https://www.youtube.com/watch?v=00VuPvEA7YA&list=PLbgAY2-LxYGq4EvICE_QDd-r9j_a_Nn4I&index=4 https://www.youtube.com/watch?v=xSKaez3aEdc&list=PLbgAY2-LxYGq4EvICE_QDd-r9j_a_Nn4I&index=5 https://www.youtube.com/watch?v=Suxaezt4Qgo&list=PLbgAY2-LxYGq4EvICE_QDd-r9j_a_Nn4I&index=6 https://www.youtube.com/watch?v=vCw8Gf77Zp0&list=PLbgAY2-LxYGq4EvICE_QDd-r9j_a_Nn4I&index=7
3. Assessment of preferred items	Parents were taught to investigate in which objects the child had greatest interest, and which activities and foods that might function as reinforcers. Chosen items were used during the training period to reinforce target behaviors. Children	6min07s	https://www.youtube.com/watch?v=2cVodkpbcOA&list=PLbgAY2-LxYGq4EvICE_QDd-r9j_a_Nn4I&index=8

	were allowed to play with the items immediately after the assessment.		
4. Errorless learning	Included instructions related to the hierarchy of prompting used in which a new skill is taught to the child by preventing his/her exposure to the error. This means using a very high level of support (prompting) to teach the target behavior, and then systematically reducing this support to a lower level as the child masters the behavior.	9min12s	https://www.youtube.com/watch?v=uVt7rRquOYo&list=PLbgAY2-LxYGq4EvICE_QDd-r9j_a_Nn4I&index=9
5. Eye contact level 1 – full physical prompt (FPP)	Parents were taught on how to use the highest level of support (full physical prompt) after saying to the child "Look at me" and when to progress to the next level.	6min08s	https://www.youtube.com/watch?v=-NsPopVWug8&list=PLbgAY2-LxYGq4EvICE_QDd-r9j_a_Nn4I&index=10
6. Eye contact level 2 – partial physical prompt (PPP)	At this second level, parents were taught on how to use the partial physical prompt after saying to the child "Look at me" and when to progress to the next level.	3min37s	https://www.youtube.com/watch?v=CmRyrh21Wi8&list=PLbgAY2-LxYGq4EvICE_QDd-r9j_a_Nn4I&index=11
7. Eye contact level 3 – gestural prompt (GP)	At this third level, parents were taught on how to use the gestural prompt after saying to the child "Look at me" and when to progress to the next level.	3min14s	https://www.youtube.com/watch?v=9psGCTliPe8&list=PLbgAY2-LxYGq4EvICE_QDd-r9j_a_Nn4I&index=12

8. Eye contact level 4 – Independence	At this level, parents were taught on how to stimulate the child to produce the behavior of eye contact independently.	3min17s	https://www.youtube.com/watch?v=4xypIhVMGt8&list=PLbgAY2-LxYGq4EvICE_QDd-r9j_a_Nn4I&index=13
9. Eye contact in a natural setting	Parents learned how to take advantage of daily situations to stimulate eye contact.	5min12s	https://www.youtube.com/watch?v=Mx9sqb7j3mc&list=PLbgAY2-LxYGq4EvICE_QDd-r9j_a_Nn4I&index=14
10. Joint attention level 1 - full physical prompt	Parents were taught on how to use the highest level of support (full physical prompt) to stimulate joint attention after putting an object of interest to the child between themself and the child. And when to progress to the next level.	5min34s	https://www.youtube.com/watch?v=duJHrtRiVEg&list=PLbgAY2-LxYGq4EvICE_QDd-r9j_a_Nn4I&index=15
11. Joint attention level 2 - partial physical prompt	Parents were taught on how to use partial physical prompt (PPP) to stimulate joint attention and when to progress to the next level.	2min47s	https://www.youtube.com/watch?v=pu1kR3sjEtc&list=PLbgAY2-LxYGq4EvICE_QDd-r9j_a_Nn4I&index=16
12. Joint attention level 3 - gestural prompt	Parents were taught on how to use gestural prompt (GP) to stimulate joint attention and when to progress to the next level.	2min55s	https://www.youtube.com/watch?v=3mve_75i49o&list=PLbgAY2-LxYGq4EvICE_QDd-r9j_a_Nn4I&index=17
13. Joint attention level 4 – independence	At this level, parents were taught on how to stimulate the child to produce the behavior of joint attention independently.	2min52s	https://www.youtube.com/watch?v=Fz5A0tNnDd0&list=PLbgAY2-LxYGq4EvICE_QDd-r9j_a_Nn4I&index=18
14. Joint attention in a natural setting	In the final video, parents learned to stimulate joint attention in a *natural setting*, such as during play time and daily activities	6min07s	https://www.youtube.com/watch?v=wHX667TSewU&list=PLbgAY2-LxYGq4EvICE_QDd-r9j_a_Nn4I&index=19
